# Authentication of Herbal Medicines *Dipsacus asper* and *Phlomoides umbrosa* Using DNA Barcodes, Chloroplast Genome, and Sequence Characterized Amplified Region (SCAR) Marker

**DOI:** 10.3390/molecules23071748

**Published:** 2018-07-17

**Authors:** Inkyu Park, Sungyu Yang, Wook Jin Kim, Pureum Noh, Hyun Oh Lee, Byeong Cheol Moon

**Affiliations:** 1 Herbal Medicine Research Division, Korea Institute of Oriental Medicine, Daejeon 305-811, Korea; pik6885@kiom.re.kr (I.P.); sgyang81@kiom.re.kr (S.Y.); ukgene@kiom.re.kr (W.J.K.); pureum322@kiom.re.kr (P.N.); 2 Phyzen Genomics Institute, Seongnam 13558, Korea; dlgusdh88@phyzen.com

**Keywords:** plant species identification, oriental medicine, plastid, dipsaci radix, phlomidis radix

## Abstract

Dried roots of *Dipsacus asper* (Caprifoliaceae) are used as important traditional herbal medicines in Korea. However, the roots are often used as a mixture or contaminated with *Dipsacus japonicus* in Korean herbal markets. Furthermore, the dried roots of *Phlomoides umbrosa* (Lamiaceae) are used indiscriminately with those of *D. asper*, with the confusing Korean names of Sok-Dan and Han-Sok-Dan for *D. asper* and *P. umbrosa*, respectively. Although *D. asper* and *P. umbrosa* are important herbal medicines, the molecular marker and genomic information available for these species are limited. In this study, we analysed DNA barcodes to distinguish among *D. asper*, *D. japonicus*, and *P. umbrosa* and sequenced the chloroplast (CP) genomes of *D. asper* and *D. japonicus*. The CP genomes of *D. asper* and *D. japonicus* were 160,530 and 160,371 bp in length, respectively, and were highly divergent from those of the other Caprifoliaceae species. Phylogenetic analysis revealed a monophyletic group within Caprifoliaceae. We also developed a novel sequence characterised amplified region (SCAR) markers to distinguish among *D. asper*, *D. japonicus*, and *P. umbrosa*. Our results provide important taxonomic, phylogenetic, and evolutionary information on the *Dipsacus* species. The SCAR markers developed here will be useful for the authentication of herbal medicines.

## 1. Introduction

Herbal medicines are widely used in oriental medicine. However, adulteration and contamination from related species, as well as from other genera, is a common problem [1]. In general, distinguishing authentic from inauthentic herbal products with the unaided eye is difficult. Thus, methods are needed to discriminate good quality herbal products from adulterated preparations.

Molecular tools are helpful in the accurate identification of species and authentication of herbal products. In plants, DNA barcoding has been used for species identification and for differentiating authentic herbal medicines from closely related species [2,3]. The nuclear ribosomal DNA (rDNA) internal transcribed spacers (ITS) region and *matK*, *rbcL*, and *psbA*-*trnH* genes have been frequently used for plant species identification, as well as for phylogenetic and evolutionary analyses [4,5]. These DNA barcodes have only been used to discriminate among closely related species in several taxa. Moreover, identifying the authentic species based on a few sites of variable nucleotide sequences is difficult. Chloroplast (CP) genome sequencing is a potential alternative to DNA barcoding.

The next generation of sequencing platforms has enabled the complete sequencing of CP genomes, which has rapidly increased. The genomic information gained from sequencing projects has been used for plant species identification, high-resolution phylogenetic analysis, marker development to differentiate between closely related species, and the evolutionary analysis of CP structural variation, genome arrangement, and gene loss, including gene transfer from the CP to the nucleus or mitochondria [6,7,8]. CPs play an important role in photosynthesis, carbon fixation, and starch and fatty acid biosynthesis in plants [9,10]. In higher plants, the CP genome ranges from 100 to 180 kb in size and consists of 110–130 genes, including protein-coding genes and those encoding ribosomal RNAs (rRNAs) and transfer RNAs (tRNAs) [6]. Comparison of CP genomes has enabled the discovery of genetically variable regions that enable the distinguishing of species [11,12]. Insertion/deletion (Indel) mutations and single nucleotide polymorphisms in CP genomes have been frequently used for species identification [11,13,14]. Furthermore, the sequence characterised amplified region (SCAR) markers have been used to distinguish authentic herbal preparations from morphologically similar plant species [15], as these are simple, highly reproducible, and cheap. Thus, this method has been frequently used for species identification and the authentication of herbal medicines [15,16,17,18]. Sequence information is obtained from DNA fingerprints or barcodes, such as the presence or absence of amplicons, using sequence-specific primers and artificial polymorphic banding patterns.

Microsatellites or simple sequence repeats (SSRs) are abundant in genomes and have been widely used in phylogenetic analysis, population genetics, and molecular breeding [19,20]. Most SSRs are repeats of A or T units and account for the AT richness of the chloroplast genomes [21,22].

Dried roots of *Dipsacus asper* Wall. ex DC., commonly known as Dipsaci Radix (“Sok-Dan” in Korean), and *Phlomoides umbrosa* (Turcz.) Kamelin and Makhm., commonly known as Phlomidis Radix (“Han-Sok-Dan” in Korean), are used as important herbal medicines in Korea [23]. In Korean herbal pharmacopoeia, only the dried roots of *D. asper* and *P. umbrosa* are described as authentic Dipsaci Radix and Phlomidis Radix, respectively [23]. Unfortunately, the dried roots of *D. japonicus* Miq. are often mixed with those of *D. asper* and misused as Dipsaci Radix in Korean herbal markets because of the morphological similarities between them. In addition, the dried roots of *P. umbrosa* are also frequently used as economically motivated adulterants of Dipsaci Radix and are used for the same clinical purpose because of the confusing name and high cost of the authentic herb [24].

However, in traditional Korean medicine, Dipsaci Radix and Phlomidis Radix are used for different clinical purposes [25]. Dipsaci Radix is used for its protective effect against liver and kidney diseases, whereas Phlomidis Radix helps to discharge phlegm and heal bruises [23]. By contrast, the currently reported pharmacological effects of Dipsaci Radix and Phlomidis Radix are completely different. Dipsaci Radix is reported to have osteo-protective effects [26,27], whereas Phlomidis Radix is reported to have anti-inflammatory effects [28,29]. Therefore, indiscriminate application of these two herbal medicines can cause unforeseen side effects and threaten their use as medicines. Moreover, differentiating herbal medicines from adulterants is important to enhance their medicinal potential and safety and to ensure proper quality control of medicinal products.

*D. asper* and *D. japonicus* belong to the Caprifoliaceae family, which consists of 800 species in 42 genera worldwide [30]. *D. asper* is mainly distributed in the highland region (1500–3700 m above sea level) of China [31], whereas *D. japonicus* is distributed in Korea and Japan [23]. In general, *D. asper* is 2 m in height and has pinnatisect leaf blades, white or lemon yellow flowers, and a white, disk-shaped calyx with papillate-setose leaves. By contrast, *D. japonicus* is 1.5 m tall and has narrowly elliptic leaf blades, purplish red flowers, and a cup-shaped calyx with non-existent papillate-setose leaves. The *Dipsacus* species have a capitula [31]. The morphological traits of *P. umbrosa* (Lamiaceae) are different from those of *Dipsacus* species. *P. umbrosa* is 0.5–1 m in height and has verticillasters, tubular calyx, and white and pink flowers [23]. Although plants of *D. asper*, *D. japonicus*, and *P. umbrosa* can be easily distinguished based on their morphological differences, the dried roots of these species are difficult to distinguish with the unaided eye. Therefore, there is a need to distinguish between authentic Dipsaci Radix and Phlomidis Radix.

In this study, we analysed universal DNA barcodes to distinguish between *D. asper*, *D. japonicus*, and *P. umbrosa*. We also sequenced and characterised the CP genomes of *D. asper* and *D. japonicus*. Comparison of their CP genome structure and phylogenetic analysis revealed genetically divergent regions in Caprifoliaceae. Furthermore, we developed novel SCAR markers for distinguishing *D. asper* and *D. japonicus* from *P. umbrosa*. Our results provide valuable genomic information for the *Dipsacus* species and an in-depth insight into the evolution within Caprifoliaceae. These data will also enable the quality control of the valuable herbal medicines, Dipsaci Radix and Phlomidis Radix.

## 2. Results and Discussion

### 2.1. Authentication of D. asper, D. japonicus, and P. umbrosa Using DNA Barcodes

To distinguish among *D. asper*, *D. japonicus*, and *P. umbrosa*, we performed DNA barcode analysis using the nuclear rDNA ITS2, *matK*, and *rbcL* regions. We sequenced a total of 12 samples among the three species and analysed the sequence alignments (Table 1). The ITS2 region and *matK* gene harbored the highest number of variable sites (ITS2 nucleotide diversity [Pi] = 0.12842; *matK* Pi = 0.10377) of the three species. The Pi value of *P. umbrosa* was dramatically different from that of *D. asper* and *D. japonicus*. A comparison between the two *Dipsacus* species revealed only three (0.2%) parsimony informative sites in *rbcL* and seven (1.8%) in ITS2. These data indicate that the universal DNA barcode regions of *D. asper* and *D. japonicus* are highly similar. DNA barcodes have been mainly used for discrimination among herbal medicine as well as for species identification [17,18]. A previous study using ITS1 and ITS2 regions showed that these sequences of *D. asper* are similar to those of *D. japonicus*, *D. chinensis*, and *D. mitis* [32]. However, these sequences are insufficient for the development of molecular markers, and *P. umbrosa* and the other DNA barcode regions were not included in this study. Although *D. asper*, *D. japonicus*, and *P. umbrosa* exhibit species-characteristic in DNA barcode regions, these cannot be applied to commercial herbal products because of the presence of adulterants. Therefore, to ensure the adequate quality control of herbal medicines, molecular markers are needed to distinguish among *D. asper*, *D. japonicus*, and *P. umbrosa*.

### 2.2. CP Genome Organisation of D. asper and D. japonicus

**Figure 1:**
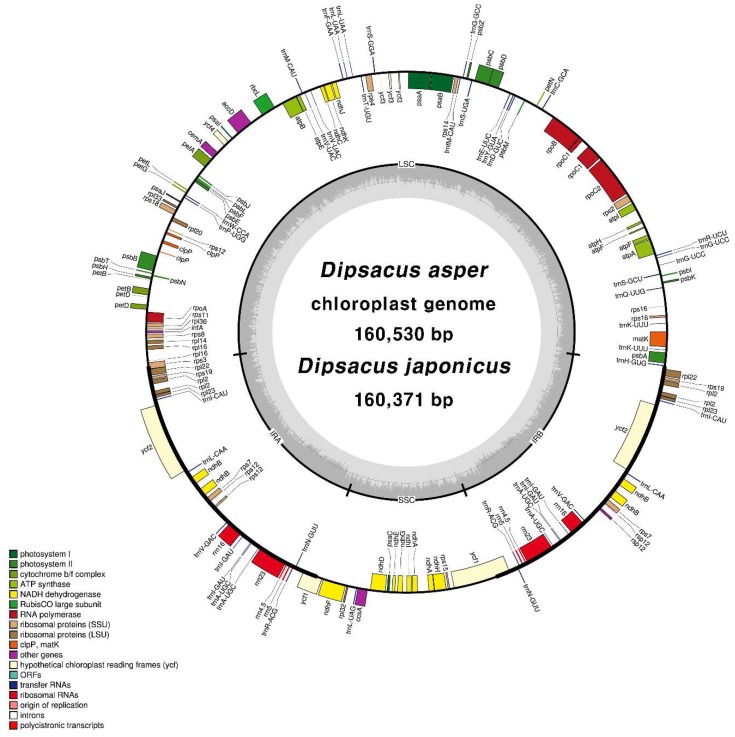
The circular gene map of *Dipsacus asper* and *D. japonicus*. The genes drawn inside the circle are transcribed clockwise, whereas those drawn outside the circle are transcribed counterclockwise. Dark gray shading inside the inner circle represents the GC content. LSC: large single copy; IR: inverted repeat; SSC: small single copy; GC: guanine-cytosine; ORF: open reading frame.

We sequenced the CP genomes of *D. asper* and *D. japonicus* to distinguish between these species. Paired-end reads of *D. asper* (2.1 Gb) and *D. japonicus* (2.2 Gb) were trimmed to obtain 1.5 and 1.6 Gb datasets, respectively. Sequencing revealed the total CP genome size of *D. asper* and *D. japonicus* as 160,530 and 160,371 bp, with 286× and 170× coverages, respectively (Table S1). Sequence reads were mapped onto the complete CP genomes (Figure S1). The CP genomes of both species were of high quality (Figure 1 and Table 2) and showed a quadripartite structure. Both chloroplast genomes were partly validated using polymerase chain reaction (PCR)-based sequencing (Table S2). Sequences of junctions between the four regions of the CP genome (large single copy (LSC)/inverted repeat (IRa), IRa/small single copy (SSC), SSC/IRb, and IRb/LSC) were aligned against the complete CP genome sequence (Table S3). The guanine-cytosine (GC) content of both species was 38.8%.

In general, the GC content of the inverted repeat (IR) regions was higher than that of the LSC and SSC regions in both species. The gene content and gene order were similar in both species. The CP genomes of both species harbored 112 unique genes, including 79 protein-coding genes, 4 rRNA genes, and 29 tRNA genes. Eighteen genes were duplicated in the IR regions (Table S4). Fifteen genes, including *rps12*, harbored a single intron, whereas *ycf3* and *clpP* carried two introns (Table S5). Alternative start codons, ACG in *ndhD* and *psbL* and GTG in *rps19*, were identified. These codons are a general phenomenon in the CP genomes of land plants [33,34,35]. We also analysed the codon usage and anticodon recognition patterns in the two *Dipsacus* species. *Dipsacus asper* and *D. japonicus* harbored 23,482 and 23,826 codons, respectively, among which codons for leucine and serine were the most abundant (Figure S2 and Table S6). Relative synonymous codon usage (RSCU) values revealed a synonymous codon bias with a high proportion of A or T at the third nucleotide position (Figure S3). This phenomenon is consistent with that found in other angiosperms [36,37,38,39]. The CP genome structures of both *Dipsacus* species were similar to that of angiosperms [21,40,41]. Thus, the two CP genome sequences had a 99.57% similarity, but no differences in the CP genome structure or gene order were detected between the two species (Table 2).

### 2.3. Analysis of Repeats in the CP Genomes of D. asper and D. japonicus

In this study, 156 and 154 SSRs were detected in the CP genomes of *D. asper* and *D. japonicus* (Figure S4). Most of the mononucleotide SSRs in the *Dipsacus* species were present in the intergenic spacer (IGS) region (Figure S4A,B). In both species, the number of SSRs per unit length was higher in the single copy regions (LSC and SSC) than in the IR regions. Seven and five species-specific SSRs were identified in *D. asper* and *D. japonicus*, respectively. We also analysed the potential of polymorphic SSRs for marker development in the two *Dipsacus* species. Twenty-seven SSRs, polymorphic between the two species comprising A or T mononucleotides, were detected (Table S7). Structural variation in CP genomes, including gene duplication, gene expansion, and genomic rearrangement, result in abundant tandem repeat sequences [42]. A total of 37 and 34 tandem repeats were detected in the CP genomes of *D. asper* and *D. japonicus*, respectively, of which 13 and 12 were short tandem repeats, respectively (Figure S5). Tandem repeats greater than 100 bp in length were abundant in both species. Most tandem repeats occurred in exons and in the IGS region present within the LSC and IR regions of the chloroplast genomes. Three palindromic repeats, 25–33 bp in length, were detected in both species (Table S8). Tandem repeats, which are variable in copy numbers between species, are often used as molecular markers [13,14].

### 2.4. Comparative Analysis of CP Genomes within Caprifoliaceae

**Figure 2:**
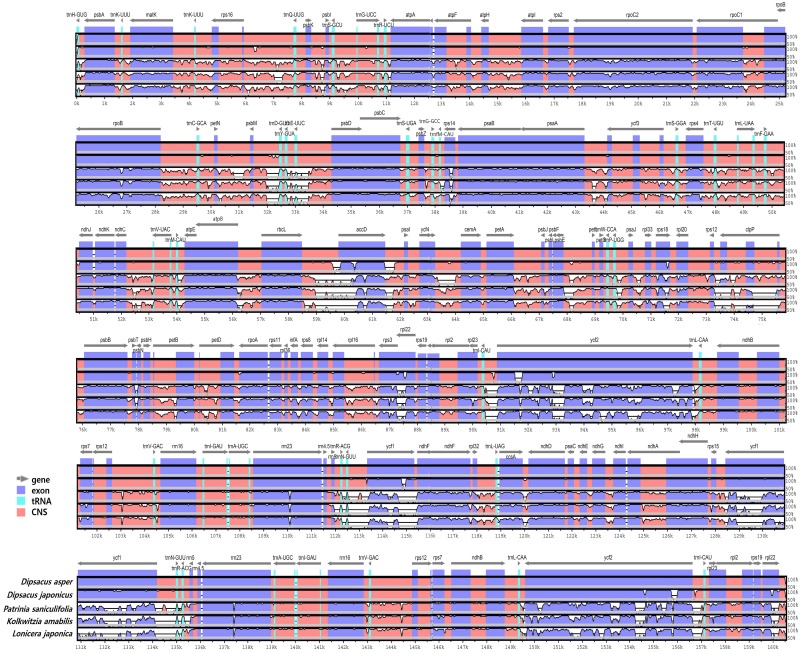
The comparative analysis of chloroplast genomes of species within the family Caprifoliaceae using mVISTA. Blue indicates conserved genes, light blue indicates tRNA and rRNA, red indicates conserved non-coding sequences (CNS), and white represents divergent regions. A cut-off of a 50% identity was used for the plots. The *Y*-axis represents 50–100% identity.

We compared the CP genome sequences of five plant species within the Caprifoliaceae family, including *D. asper*, *D. japonicus*, *Patrinia saniculifolia*, *Kolkwitzia amabilis*, and *Lonicera japonica*, to identify divergent regions (Figure 2). The CP genomes of *D. asper* and *D. japonicus* were highly conserved in the coding and non-coding regions, except for the *accD* and *ycf2* genes, but were highly divergent from the other Caprifoliaceae species in the coding region. The divergence patterns of *P. saniculifolia*, *K. amabilis*, and *L. japonica* were similar to those of *D. asper* and *D. japonicus*. We compared the LSC, IRa, SSC, and IRb boundaries in the five species (Figure S6). The structure of the IR region was highly similar in the two *Dipsacus* species. The *rpl3* gene extended into the IRa region in *D. asper*, *D. japonicus*, *K. amabilis*, and *L. japonica*. The *ycf1* genes were located at the IRa/SSC and SSC/IRb junctions in *D. asper*, *D. japonicus*, and *P. saniculifolia*. Overall, the CP genomes of the five species were highly divergent in the IR regions. Contraction and expansion of the IR regions due to genomic rearrangement is a common phenomenon in angiosperms [43]. Further investigations are needed to dissect the structural variation in the chloroplast genomes and to understand the evolutionary relationships within Caprifoliaceae.

**Figure 3:**
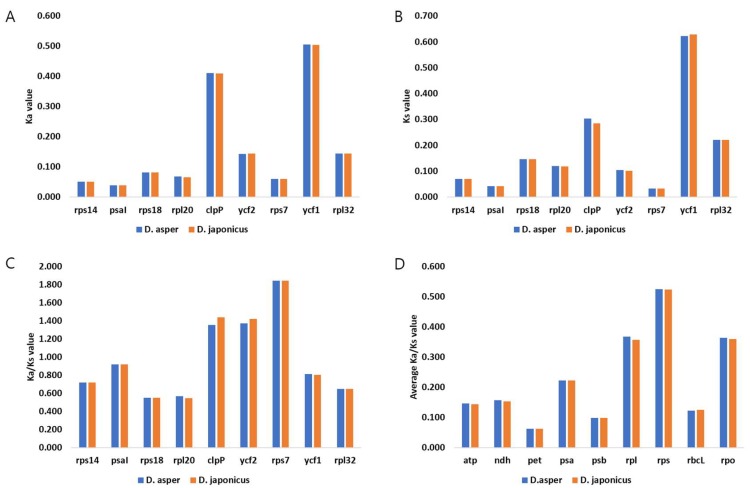
The analysis of Ka, Ks, and Ka/Ks values of the chloroplast genomes of *D. asper*, *D. japonicus*, and *L. japonica*. The ratio of non-synonymous substitution rate (Ka) to synonymous substitution rate (Ks) was calculated for 78 conserved protein-coding genes. (**A**) Ka values of genes with Ka/Ks > 0.5. (**B**) Ks value of genes with Ka/Ks > 0.5. (**C**) Ka/Ks values of specific genes. (**D**) Average Ka/Ks values of gene groups.

The ratio of non-synonymous to synonymous mutation rates (Ka/Ks) was used to understand gene evolution. Adaptive evolution in response to the environment occurs when the genes are under positive selection [37,44,45]. We calculated the Ka/Ks ratios of 78 collinear protein-coding genes in the two *Dipsacus* species against those in *L. japonica* to identify the selection pressure on these genes in Caprifoliaceae (Figure 3). The most conserved genes appeared to be undergoing purifying selection, as the Ka/Ks ratio was less than 0.001. No significant differences were detected in the Ka/Ks ratios among the LSC, IR, and SSC regions. The Ka/Ks ratios of the two *Dipsacus* species varied from 0.001 to 1.843 (average = 0.284). The Ka/Ks ratio of the *rps* genes encoding the small ribosomal subunits was 0.524, indicating a relaxed selection. Among the *rps* genes, *rps7* showed the highest Ka/Ks ratio (1.843), indicating a positive selection. The *rps7* gene encodes the ribosomal protein S7 and has been reported under a positive selection in the *Salix* species (Salicaceae). The *clpP* and *ycf2* genes also showed positive selection (Ka/Ks > 1); these genes have been frequently reported as lost or highly variable [6,10,46]. The *clpP* gene encodes a protein involved in translation and protein modification, whereas *ycf2* has an unknown function [6]. In this study, *clpP* and *ycf2* showed a weak selection during the adaptive evolution in Caprifoliaceae. Taken together, we suggest that *rps7*, *ycf2*, and *clpP* evolved rapidly in Caprifoliaceae. These genes were found from other CP genomes [47,48]. We propose that the positive selection of these genes undergo essential adaptations to the environment.

**Figure 4:**
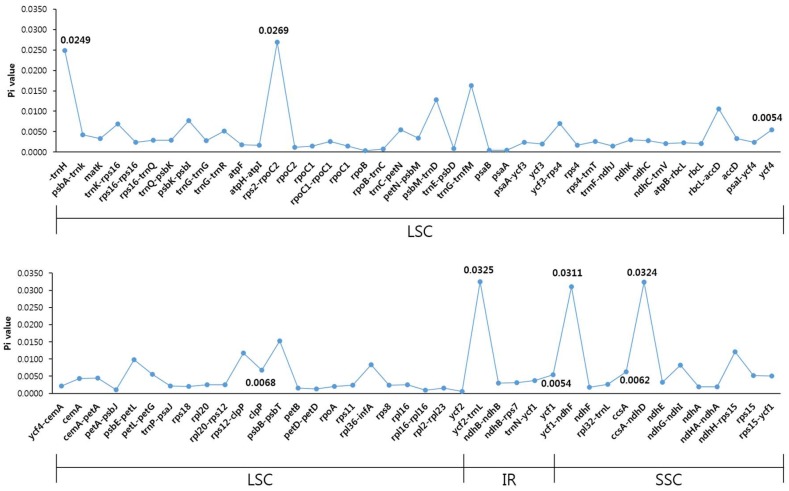
The comparison of nucleotide diversity (Pi) between the chloroplast genomes of *D. asper* and *D. japonicus*.

To examine divergence at the sequence level in *D. asper* and *D. japonicus*, we estimated the Pi (Figure 4). A total of 81 regions with a Pi value greater than zero were classified as coding, non-coding, and IGS. Single copy regions (LSC and SSC) were more variable than the IR regions. The average Pi values for the single copy regions was 000054 and 0.0065 for the IR regions. Genic regions were more conserved than the IGS region. The *ycf2*-*trnL* region showed the highest Pi value of 0.0325. Among the protein-coding genes, *clpP* showed the highest Pi value and was highly variable in the two *Dipsacus* species, indicating the positive selection in Caprifoliaceae. Although the CP genomes of *D. asper* and *D. japonicus* showed a highly conserved structure, the intergenic regions and a few coding regions diverged. In the chloroplast genomes of angiosperms, the intergenic regions have a higher representation than the genic regions [49].

**Figure 5:**
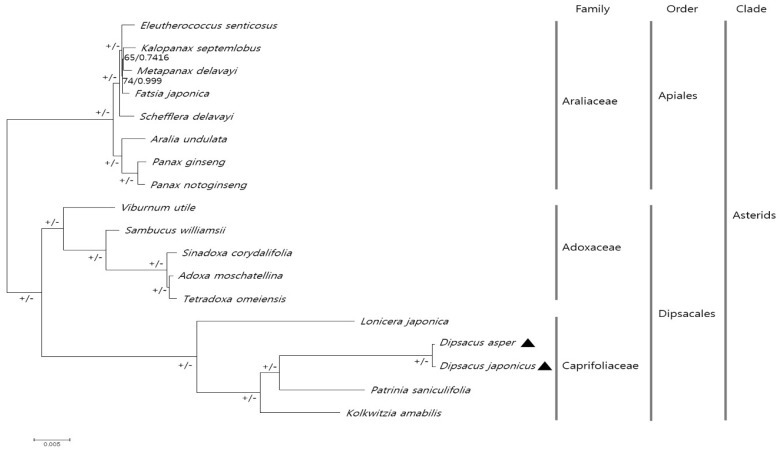
The phylogenetic analysis of 69 protein-coding genes in 18 species, including *D. asper* and *D. japonicus*, using the maximum likelihood (ML) method and Bayesian posterior probabilities (PP). The ML topology is shown with the ML bootstrap support values and Bayesian PP given at each node. A total of 100% bootstrap and 1.0 PP support are indicated above each node using + and −, respectively. The black triangles indicate the CP genomes of *D. asper* and *D. japonicus* examined in this study.

The CP genomes have been successfully used in numerous phylogenetic studies of angiosperms. Using the chloroplast genomes is advantageous, as these are more accurate and have a greater resolution than a few cp loci and nuclear rDNA ITS regions [50,51]. We investigated the phylogenetic relationship among 18 species in the families Caprifoliaceae and Adoxaceae within the order Dipsacales, and in the family Araliaceae within the order Apiales using the maximum likelihood (ML) and Bayesian inference (BI). Sequences of 69 CP protein-coding genes from all 18 species were aligned (alignment length = 58,243 bp) (Figure 5). Most nodes showed 100% ML bootstrap values and 1.0 BI posterior probabilities, except for two nodes in Araliaceae. All 18 species have been shown to cluster according to the APG IV system [52]. *P. saniculifolia* formed a sister group with two *Dipsacus* species, and. *L. japonica* showed a distinct phylogenetic relationship to the other four species in Caprifoliaceae. The phylogenic tree reconstructed in this study was clearly consistent with those in previous studies [53,54,55]. Previous studies used the ITS/ITS2 region or other CP loci to understand the phylogenetic relationship among the Caprifoliaceae or Dipsacales species [53,54,55]. Despite the few CP genomes applied in this study, this is the first report of phylogenetic analysis of the genus *Dipsacus* using protein-coding genes in the CP genome. These data provide an improved phylogenetic relationship among the species within Caprifoliaceae, supported by strong bootstrap values.

### 2.5. Authentication of D. asper, D. japonicus, and P. umbrosa Using SCAR Markers

**Figure 6:**
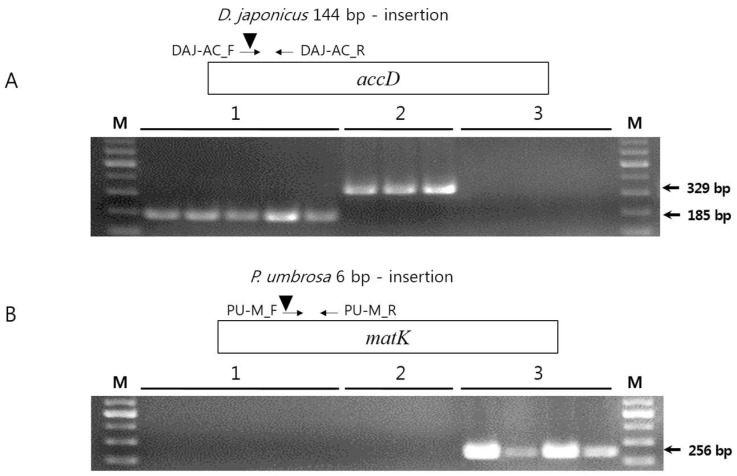
The gel images of CP DNA of *D. asper*, *D. japonicus*, and *P. umbrosa* amplified using sequence characterised amplified region (SCAR) markers. Twelve individual plants were used in this study using (**A**) DAJ-AC and (**B**) PU-M primers. Details of the germplasm are provided in Supplementary Table S10. (**1**) *D. asper*, (**2**) *D. japonicus*, (**3**) *P. umbrosa*.

The gene region is more stable than the IGS region for the development of molecular markers. In this study, indels were detected in the *accD* gene of both *Dipsacus* species and in the *matK* gene of *P. umbrosa*. We used these indels for the development of SCAR markers to distinguish between *D. asper* and *D. japonicus*. The primer pairs DAJ-AC_F/DAJ-AC_R and PU-M_F/PU-M_R were used to amplify the *accD* and *matK* genes from the two *Dipsacus* species and *P. umbrosa*, respectively (Table S9). We also tested these markers on 12 individual plants, including five *D. asper*, three *D. japonicus*, and four *P. umbrosa* individual plants, obtained from the Korea Institute of Oriental Medicine (KIOM) (Figure 6 and Table S10). The DAJ-AC primers amplified 185 and 329 bp amplicons from *D. asper* and *D. japonicus*, respectively, but showed no amplification from *P. umbrosa*. In contrast, the PU-M primers amplified a 256 bp fragment from *P. umbrosa* but not from the *Dipsacus* species.

SCAR markers are a valuable tool for species identification and have been used to discriminate herbal medicines from adulterants. Moreover, SCAR markers are reliable and easy to use, requiring only PCR and gel electrophoresis [18]. A CP-specific SCAR marker was previously used to distinguish *Aconitum coreanum* from its closely related species on the basis of a 6 bp insertion in its CP genome [16]. The nuclear rDNA ITS barcode region has been frequently used for the development of SCAR markers as it is highly variable in sequence. SCAR markers have been used to differentiate valuable herbal plants, including *Aralia continentalis* and *Angelica biserrata*, from adulterants [17]. Therefore, SCAR markers are a powerful molecular tool for the identification of herbal medicines. The novel SCAR markers developed in this study will be useful for the identification of *D. asper*, *D. japonicus*, and *P. umbrosa* and the authentication of herbal medicines.

## 3. Materials and Methods

### 3.1. Plant Materials

Fresh leaves of *D. asper* (36°43′35.9″ N and 127°27′38.0″ E) and *D. japonicus* (37°14′04.5″ N and 128°56′57.0″ E) were collected from their native habitats in Korea. All samples were assigned unique identification numbers and registered with the Korean Herbarium of Standard Herbal Resources (Index Herbarium code KIOM) at the KIOM. Details of these samples are listed in the Supplementary Table S10.

### 3.2. Universal DNA Barcode Analysis for D. asper, D. japonicus, and P. umbrosa

Universal DNA barcodes, including ITS2, *matK*, and *rbcL*, were PCR amplified using ITS2-s2f/ITS4, matK-AF/matK-8R, and rbcL-N/rbcL-R primer pairs, respectively, from the nuclear and CP of *D. asper*, *D. japonicus*, and *P. umbrosa*, using previously described amplification parameters [56,57,58]. The amplified DNA fragments were extracted from the agarose gel using a gel extraction kit, cloned into a pGEM-T Easy vector (Promega, Madison, WI, USA), and sequenced on an ABI 3730 DNA Analyzer (Applied Biosystems Inc., Foster City, CA, USA) [17,18]. The obtained sequences were aligned and edited using BioEdit ver. 7.2.5 [59]. Parsimony informative sites and Pi values were calculated using DnaSP ver. 5.1 [60].

### 3.3. Genome Sequencing and Assembly

DNA was extracted using DNeasy Plant Maxi kit (Qiagen, Valencia, CA, USA), according to the manufacturer’s instructions. Illumina short-insert paired-end sequencing libraries were constructed and sequenced using the NextSeq platform (Illumina, San Diego, CA, USA). CP genome sequences were determined from the de novo assembly of low-coverage whole-genome sequences. Trimmed paired-end reads (Phred score ≥ 20) were assembled using the CLC genome assembler ver. 4.06 beta (CLC Inc., Rarhus, Denmark) with the default parameters. Principal contigs representing the chloroplast genome were retrieved from the total collection of contigs using Nucmer [61] and aligned with the chloroplast genome sequence of *Ilex wilsonii* (KX426471) as a reference. A de novo SOAP gap closer was performed to fill the gaps based on aligned paired-end reads [62]. PCR-based sequencing was used to validate the sequences of the four chloroplast junction regions, LSC/IR, IR/SSC, SSC/IR, and IR/LSC, using the primers listed in Supplementary Table S2. Finally, the CP genome sequence reads were mapped onto the complete genomes using a Burrows-Wheel Aligner ver. 0.7.25 [63].

### 3.4. Genome Annotation and Comparative Analysis

Gene annotation of the CP genomes of *D. asper* and *D. japonicus* was performed using GeSeq [64] and the annotation results were concatenated using an in-house pipeline. Protein-coding sequences were manually curated and confirmed using Artemis [65] and checked against the protein database of the National Center for Biotechnology Information (NCBI). Sequences of the tRNA genes were confirmed using tRNAscan-SE 1.21 [66], and those of the IR regions were confirmed using the IR finder and RepEx [67]. Circular maps of the two Dipsacus CP genomes were obtained using OGDRAW [68]. The GC content and RSCU values were analysed using the MEGA6 software [69]. Sequences of the LSC/IR, IR/SSC, SSC/IR, and IR/LSC junctions of the completed CP genomes were validated via PCR-based sequencing using the primers listed in Tables S2 and S3. NCBI accession numbers of the CP genome sequences of *D. asper* and *D. japonicus* are MH074864 and MH074865, respectively. The CP genomes of *D. asper*, *D. japonicus*, *Patrinia saniculifolia* (NC_036835.1), *Kolkwitzia amabilis* (NC_029874.1), and *Lonicera japonica* (NC_026839.1) were compared using mVISTA in the Shuffle-LAGAN mode, with the CP genome of *D. asper* as a reference. The Pi values for *D. asper* and *D. japonicus* were calculated using DnaSP ver. 5.1 [60]. The step size was set to 200 bp. To analyse the synonymous (Ks) and non-synonymous substitution rates, shared functional protein gene sequences and amino acid sequences were used for calculating Ka and Ks values using PAML with the yn00 program [70].

### 3.5. Repeat Analysis

SSRs in the CP genomes of the two *Dipsacus* species were detected using MISA [71], with the minimum number of repeats set to 10, 5, 4, 3, 3, and 3 for mono-, di-, tri-, tetra-, penta-, and hexa-nucleotides, respectively. The minimum alignment score and maximum period size of tandem and palindromic repeats for 20 or more bp were set at 50 and 500, respectively, and the identity of repeats was set to 90% or greater [72,73].

### 3.6. Phylogenetic Analysis

The CP genomes of 18 species belonging to the families Caprifoliaceae, Adoxaceae, and Araliaceae were subjected to phylogenetic analyses. Of these, the CP genome sequences of 16 species were downloaded from NCBI (Supplementary Table S11). To construct the phylogenetic tree, sequences of 69 protein-coding genes were aligned using MAFFT [74] and manually adjusted using BioEdit [59]. The best-fitting model of nucleotide substitutions was determined according to the Akaike Information Criterion (AIC) in JModeltest ver. 2.1.10 [75]. The GTR+I+G model was used in both. The maximum likelihood method was used to construct the phylogenetic tree in MEGA6 [69], with 1000 bootstrap replicates. Bayesian inference analyses were conducted using MrBayes ver. 3.2.2 [76], with two independent runs and four chains using the Markov chain Monte Carlo (MCMC), and simultaneous runs for one million generations. Each chain started with a random tree with default priors. Phylogenetic trees were sampled every 1,000,000 generations with the first 25% discarded as burn-in. The consensus tree was determined based on the 50% majority rule to estimate the posterior probabilities.

### 3.7. Development of SCAR Markers for D. asper, D. japonicus, and P. umbrosa

To develop the SCAR markers, primers flanking the variable region in the *accD* gene of *D. asper* and *D. japonicus* were designed using the Primer-BLAST (NCBI) [77]. The genomic DNA (20 ng) of *D. asper*, *D. japonicus*, and *P. umbrosa* was PCR amplified in a 20 L volume using 10 pmol primers. SCAR markers were amplified using species-specific primers (DAJ-AC and PU-M, Table S9), according to the following conditions: DAJ-AC, 95 °C for 2 min, followed by 35 cycles at 95 °C for 30 s, 63 °C for 30 s, and 72 °C for 30 s, and a final extension at 72 °C for 5 min; PU-M, 95 °C for 2 min, followed by 35 cycles at 95 °C for 50 s, 63 °C for 30 s, and 72 °C for 50 s, and a final extension at 72 °C for 5 min. The PCR products were verified by gel electrophoresis on a 2% agarose gel for 40 min at 150 V.

## 4. Conclusions

In this study, we performed a DNA barcoding analysis of *D. asper*, *D. japonicus* and *P. umbrosa* which are important herbal plants. We sequenced the chloroplast genomes of *D. asper*, *D. japonicus*. *D. asper* and *D. japonicus*, which exhibited slightly different parsimony informative sites in the ITS2, *matK*, and *rbcL* regions. The CP genome structure of these two *Dipsacus* species was highly conserved but was different from that of the other Caprifoliaceae species. We also investigated the phylogenetic relationship among the Caprifoliaceae species; this is the first report of phylogenetic analysis of the two *Dipsacus* species based on CP genomes. Additionally, we developed novel SCAR markers, DAJ-AC and PU-M, for the identification of the authentic herbal medicines, Dipsaci Radix and Phlomidis Radix. Taken together, these data facilitate the distinguishing of *D. asper*, *D. japonicus*, and *P. umbrosa* from adulterants in the herbal medicine market and enable the commercial use of the Dipsaci Radix and Phlomidis Radix. Furthermore, these results provide valuable information on the CP genomes and evolution of species within Caprifoliaceae.

## Figures and Tables

**Table 1:** The analysis of universal DNA barcodes in *Dipsacus asper*, *D. japonicus*, and *P. umbrosa*.

Species	DNA	AL ^1^ Length (bp)	Parsimony Informative Sites		Variable Sites		Nucleotide Diversity (Pi)	No. of Indels	No. of Haplotypes
			No.	%	No.	%			
*D. asper* vs. *D. japonicus* vs. *P. umbrosa*	ITS2	390	91	23.3	145	37.2	0.12842	8	8
	*matK*	1276	234	18.3	242	19.0	0.10377	2	8
	*rbcL*	1511	106	7.0	113	7.5	0.0394	1	6
*D. asper* vs. *D. japonicus*	ITS2	379	7	1.8	9	2.4	0.00718	0	4
	*matK*	1264	5	0.4	8	0.6	0.00332	0	4
	*rbcL*	1501	3	0.2	6	0.4	0.00233	0	4

^1^ AL length: alignment length.

**Table 2:** The characteristics of the chloroplast (CP) genomes of *D. asper* and *D. japonicus*.

Characteristic	*D. asper*	*D. japonicus*
Accession number	MH074864	MH074865
Total CP genome size (bp)	160,530	160,371
LSC region (bp)	86,979	87,193
IR region (bp)	27,821	27,664
SSC region (bp)	17,909	17,850
Number of genes	112	112
Protein-coding genes	79	79
rRNA	4	4
tRNA	29	29
Introns (bp)	14,368	14,392
Intergenic spacers (bp)	50,578	51,526
GC content (%)	38.8	38.8
LSC (%)	37.2	37.1
IR (%)	42.8	42.9
SSC (%)	34.2	34.2

CP: Chloroplast; LSC: Large single copy; IR: Inverted repeat; SSC: Small single copy.

## Supplementary Materials

The following are available online. Figure S1: Distribution of paired-end reads mapped onto the complete chloroplast genomes of *Dipsacus asper* and *D. japonicus*, Figure S2: Frequencies of amino acid in two *Dipsacus* protein-coding sequences, Figure S3: Relative synonymous codon usage (RSCU) values for 20 amino acids and the stop codon in 78 protein-coding genes present in the chloroplast genomes of *D. asper* and *D. japonicus*, Figure S4: Distribution of simple sequence repeats (SSRs) in the chloroplast genomes of *D. asper* and *D. japonicus*. Number of (A) SSRs in exons, introns, and intergenic spacer (IGS) regions; (B) different SSR types; (C) SSRs per unit length of the chloroplast genomes; and (D) common and species-specific SSRs, Figure S5: Analysis of tandem repeats in the chloroplast genomes of *D. asper* and *D. japonicus*. Distribution of (A) tandem repeats of different lengths and (B) tandem repeats in different regions of the chloroplast genomes. Number of (C) tandem repeats in the IGS region, exons, and introns; and (D) common and species-specific tandem repeats, Figure S6: Comparison of junctions between the large single copy (LSC) region, inverted repeat (IR) regions (IRa and IRb), and small single copy (SSC) region in the chloroplast genomes of the Caprifoliaceae species, *Dipsacus asper*, *Dipsacus japonicus*, *Patrinia saniculifolia*, *Kolkwitzia amabilis*, and *Lonicera japonica*, Table S1: Details of the raw sequence reads and chloroplast genome assembly of the two *Dipsacus* species, Table S2: List of primers used for the validation of chloroplast genomes of *D. asper* and *D. japonicus*, Table S3: Polymerase chain reaction (PCR)-based sequence validation of junctions between the large single copy (LSC), small single copy (SSC), and inverted repeat (IRa and IRb) regions in the chloroplast genomes of *D. asper* and *D. japonicus*, Table S4: List of genes identified in the chloroplast genomes of *D. asper* and *D. japonicus* along with the encoded proteins, Table S5: Location and sizes of genes in the chloroplast genomes of *D. asper* and *D. japonicus*, Table S6: Distribution of amino acids in the chloroplast genomes of *D. asper* and *D. japonicus*, Table S7: Details of polymorphic simple sequence repeats (SSRs) in the chloroplast genomes of *D. asper* and *D. japonicus*, Table S8: Details of palindromic repeats in the chloroplast genomes of *D. asper* and *D. japonicus*, Table S9: List of primers used for the amplification of sequence characterised amplified region (SCAR) markers, Table S10: Details of germplasm used in this study, Table S11: List of chloroplast genomes downloaded from National Center for Biotechnology Information (NCBI) for phylogenetic analysis.
