# Supplementary material for: Authentication of Herbal Medicines Dipsacus asper and Phlomoides umbrosa Using DNA Barcodes, Chloroplast Genome, and Sequence Characterized Amplified Region (SCAR) Marker
Source: Molecules. 2018 Jul 17;23(7):1748. doi: 10.3390/molecules23071748 (PMC6099718; doi:10.3390/molecules23071748)
Supplement: Supplementary file 1 [file molecules-23-01748-s001.pdf]

molecules

MDPI

Supplementary Materials: Authentication of Herbal Medicines *Dipsacus asper* and *Phlomoides umbrosa* using DNA barcode, Chloroplast Genome, and Sequence Characterized Amplified Region (SCAR) marker

Inkyu Park, Sungyu Yang, Wook Jin Kim, Pureum Noh, and Byeong Cheol Moon*

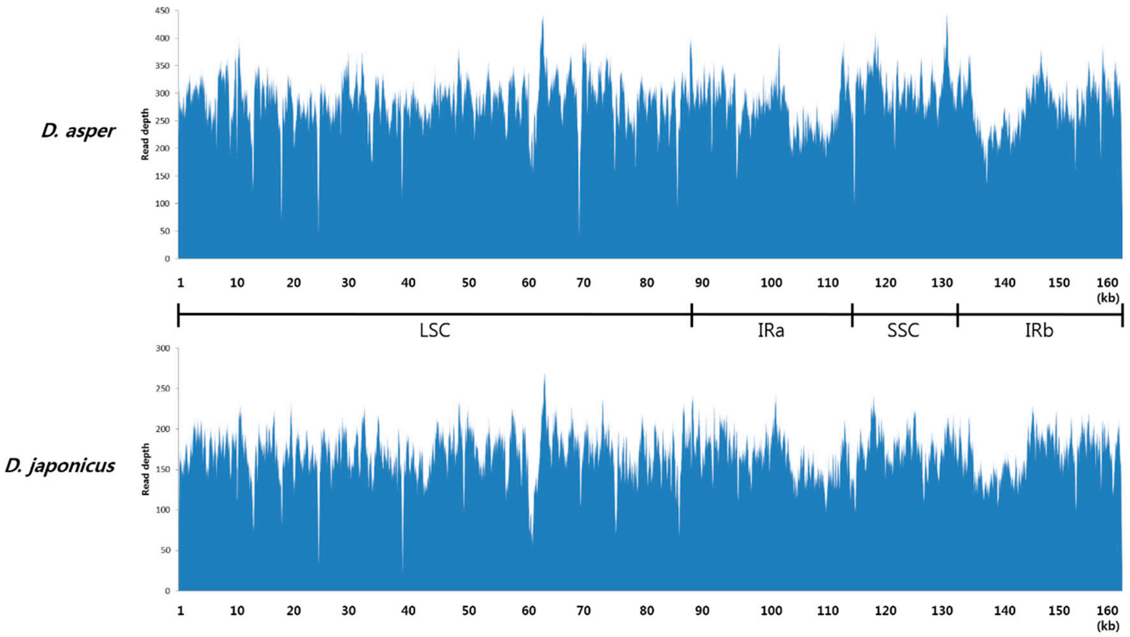

Figure S1: Distribution of paired-end reads mapped onto the complete chloroplast genomes of *Dipsacus asper* and *D. japonicus*.

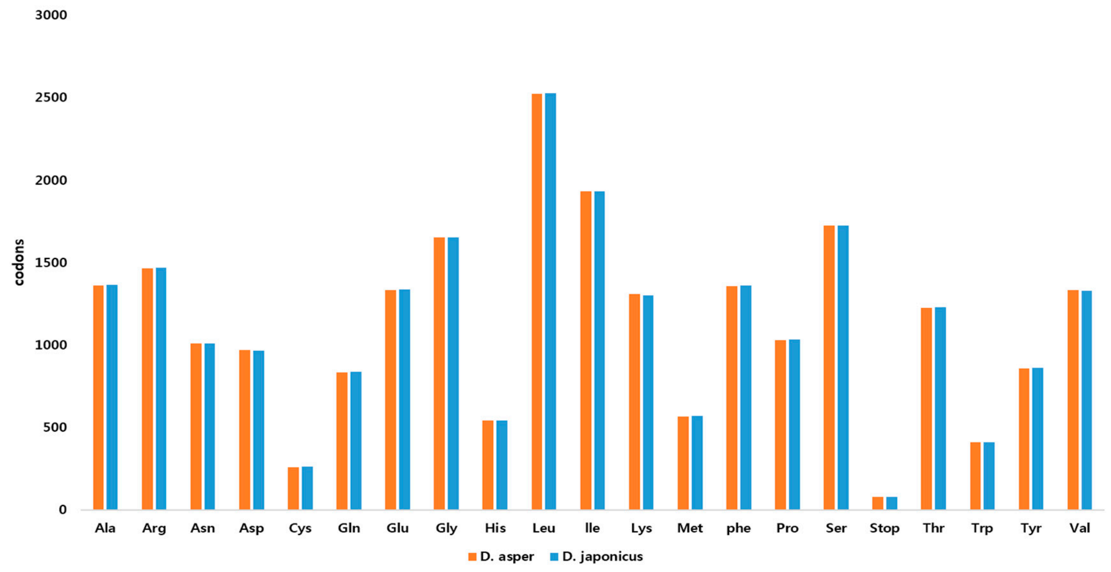

Figure S2: Frequencies of amino acid in two *Dipsacus* protein-coding sequences.

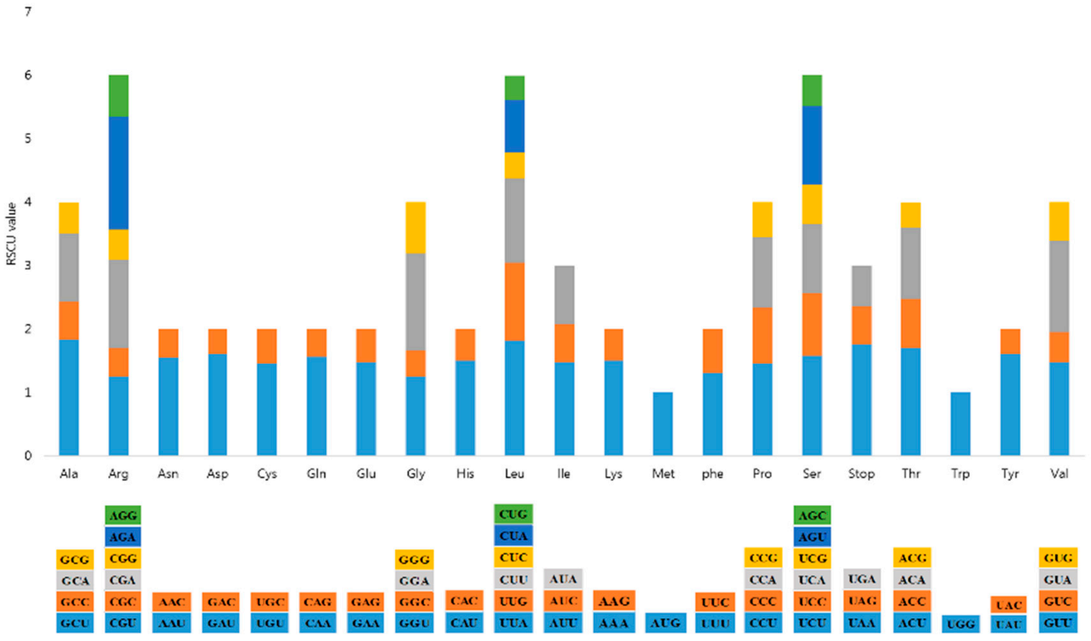

Figure S3: Relative synonymous codon usage (RSCU) values for 20 amino acids and the stop codon in 78 protein-coding genes present in the chloroplast genomes of *D. asper* and *D. japonicus*.

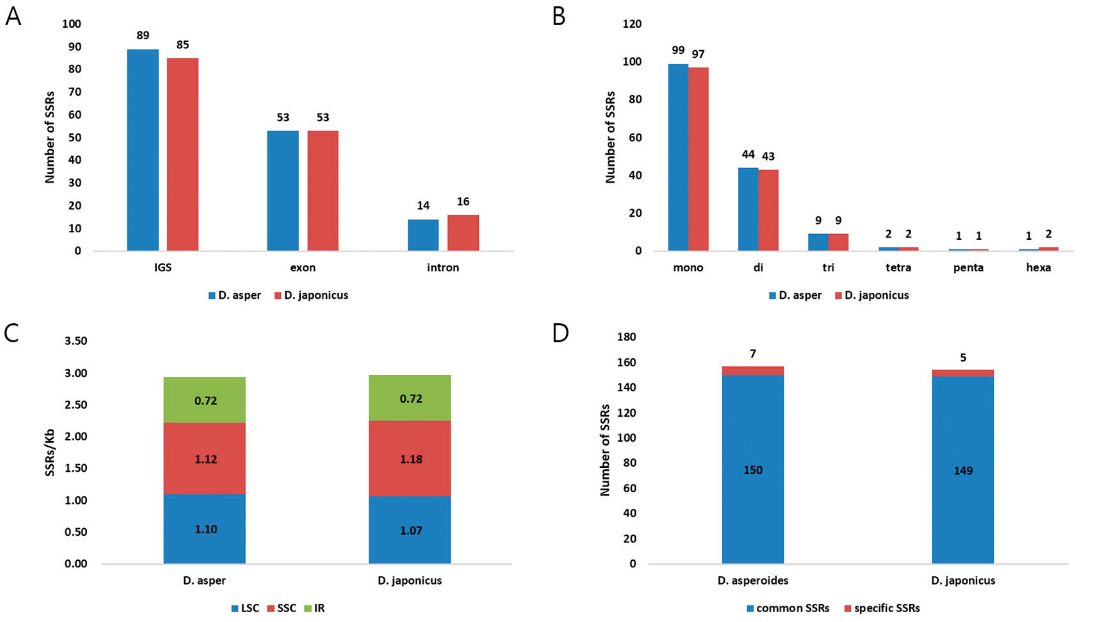

Figure S4: Distribution of simple sequence repeats (SSRs) in the chloroplast genomes of *D. asper* and *D. japonicus*. (A) Number of SSRs in exons, introns, and intergenic spacer (IGS) regions. (B) Number of different SSR types. (C) Number of SSRs per unit length of the chloroplast genomes. (D) Number of common and species-specific SSRs.

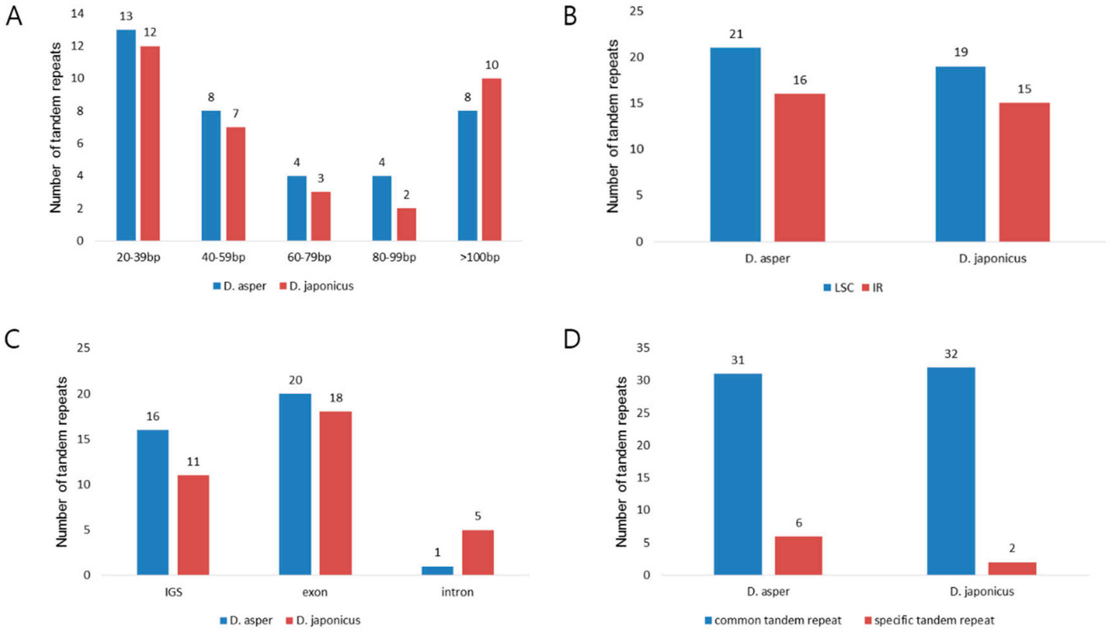

Figure S5: Analysis of tandem repeats in the chloroplast genomes of *D. asper* and *D. japonicus*. (A) Distribution of tandem repeats of different lengths. (B) Distribution of tandem repeats in different regions of the chloroplast genomes. (C) Number of tandem repeats in the IGS region, exons, and introns. (D) Number of common and species-specific tandem repeats.

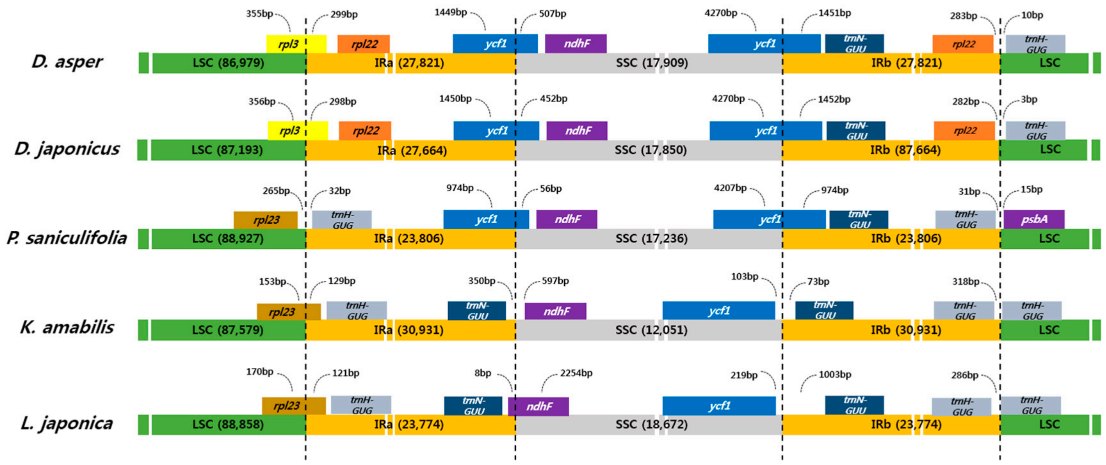

Figure S6: Comparison of junctions between the large single copy (LSC) region, inverted repeat (IR) regions (IRa and IRb), and small single copy (SSC) region in the chloroplast genomes of the Caprifoliaceae species, *Dipsacus asper*, *Dipsacus japonicus*, *Patrinia saniculifolia*, *Kolkwitzia amabilis*, and *Lonicera japonica*.

Table S1: Details of the raw sequence reads and chloroplast genome assembly of the two *Dipsacus* species.

	D. asper	*D. japonicus*
Input reads	7,293,588	7,379,022
Trimmed reads	6,359,030	6,479,504
Total raw bases (bp)	2,179,118,146	2,205,117,846
Trimmed bases (bp)	1,577,435,941	1,616,928,641
Aligned reads	189,350	112,697
Coverage (X)	286	170
Chloroplast genome size (bp)	160,530	160,371

Table S2: List of primers used for the validation of chloroplast genomes of *D. asper* and *D. japonicus*.

Primer name	Primer sequence (5′→3′)	Product length in *D. asper*
DIPLI_F	GGTTGTGCAAACCAAACGGA	647
DIPLI_R	TCAAATTCGTGGGCGTTCCT	
DIPIS_F	TGGCAAGAGAAATTACGCCCT	398
DIPIS_R	TGAAAAGCCCACATACGACGA	
DIPSI_F	GCTAGATGCAGAGGCGCATA	808
DIPSI_R	AAGATGGCAGGCGGCTTTAT	
DIPIL_F	ATCAAATTCGTGGGCGTTCC	943
DIPIL_R	GCCAAGTGGATCAAGGCAGT	

Table S3: PCR-based sequence validation of junctions between the large single copy (LSC), small single copy (SSC), and inverted repeat (IRa and IRb) regions in the chloroplast genomes of *D. asper* and *D. japonicus*.

Species	Junction	PCR product length (bp)	Chloroplast genome size (bp)	Start position	End position	Number of identical nucleotides	Percent identity
D. asper	LSC/IRa	647	160,530	87,507	87,847	647	100
	IRa/SSC	398		115,171	115,568	398	100
	SSC/IRb	808		133,893	134,700	808	100
	IRb/LSC	943		160,163	74	943	100
D. japonicus	LSC/IRa	617	160,371	87,414	88,030	617	100
	IRa/SSC	398		115,173	115,570	398	100
	SSC/IRb	808		133,892	134,696	808	100
	IRb/LSC	905		159,535	67	905	100

Table S4: List of genes identified in the chloroplast genomes of *D. asper* and *D. japonicus* along with the encoded proteins.

Proteins	Gene names
Photosystem I	*psaA, psaB, psaC, psaI, psaJ, ycf3*[2], *ycf4*
Photosystem II	*psbA, psaB, psaC, psaD, psaE, psaF, psaH, psaI, psaJ, psaK, psaL, psaM, psaN, psaT, psaZ*
Cytochrome *b6/f*	*petA, petB*[1], *petD*[1], *petG, petL, petN*
ATP synthase	*atpA, atpB, atpE, atpF*[1], *atpH, atpI*
Rubisco	*rbcL*
NADH oxidoreductase	*ndhA*[1], *ndhB*[1,3], *ndhC, ndhD, ndhE, ndhF, ndhG, ndhH*[3], *ndhI, ndhJ, ndhK*
Large subunit ribosomal proteins	*rpl2*[1,3], *rpl14, rpl16*[1], *rpl20, rpl22, rpl23*[3], *rpl32, rpl33, rpl36*
Small subunit ribosomal proteins	*rps2, rps3, rps4, rps7*[3], *rps8, rps11, rps12*[2,3,4], *rps14, rps15, rps16, rps18, rps19*
RNA polymerase	*rpoA, rpoB, rpoC1*[1], *rpoC2*
Unknown function	*ycf1*[3], *ycf2*[3]
Miscellaneous	*accD, ccsA, cemA, clpP*[2], *matK*
Ribosomal RNAs	*rrn16*[3], *rrn23*[3], *rrn4.5*[3], *rrn5*[3]
Transfer RNAs	*trnA-UGC*[1,3], *trnC-GCA, trnD-GUC, trnE-UUC, trnF-GAA, trnfM-CAU, trnG-GCC, trnG-UCC*[1], *trnH-GUG, trnI-CAU*[3], *trnI-GAU*[1,3], *trnK-UUU*[1], *trnL-CAA*[3], *trnL-UAA, trnL-UAG, trnM-CAU, trnN-GUU*[3], *trnP-UGG, trnQ-UUG, trnR-ACG*[3], *trnR-UCU, trnS-GCU, trnS-GGA, trnS-UGA, trnT-UGU, trnV-GAC, trnV-UAC, trnW-CCA, trnY-GUA*

[1] Genes containing a single intron.

[2] Genes containing two introns.

[3] Genes with two copies in the IR regions.

[4] Trans-splicing genes.

Table S5: Location and sizes of genes in the chloroplast genomes of *D. asper* and *D. japonicus*.

	Gene	Location	Exon I[1]	Intron I[1]	Exon II[1]	Intron II[1]	Exon III[1]
1	*trnK-UUU*	LSC	37	2565*	35		
2	*rps16*	LSC	40	853 (852)	236		
3	*trnG-UCC*	LSC	26	717 (725)	47		
4	*atpF*	LSC	144	699	411		
5	*rpoC1*	LSC	456	786 (791)	1611		
6	*ycf3*	LSC	126	737	228	753	153
7	*trnL-UAA*	LSC	37	513 (512)	50		
8	*trnV-UAC*	LSC	39	577	37		
9	*rps12*	LSC	114		232		26
10	*clpP*	LSC	71	834 (839)	292	679 (681)	231
11	*petB*	LSC	6	811	642		
12	*petD*	LSC	8	750	475		
13	*rpl16*	LSC	9	1092 (1097)	399		
14	*ndhB*	IR	777	681	756		
15	*trnI-GAU*	IR	42	943	35		
16	*trnA-UGC*	IR	38	808	35		
17	*ndhA*	SSC	553 (552)	1084 (1085)	539 (540)		

[1] Numbers represent the gene size (bp) in *D. asper*; numbers in parentheses represent the gene size in *D. japonicus*. * The intron length of *trnK-UUU* include *matK*.

Table S6: Distribution of amino acids in the chloroplast genomes of *D. asper* and *D. japonicus*.

Amino acid	*D. asper*		*D. japonicus*		tRNA
	Count	RSCU[2]	Count	RSCU[1]	
Phe	877	1.29	875	1.29	trnF-GAA
Phe	485	0.71	484	0.71	trnL-UAA
Leu	750	1.78	748	1.78	trnL-CAA
Leu	534	1.27	536	1.27	
Leu	533	1.26	530	1.26	
Leu	197	0.47	199	0.47	trnL-UAG
Leu	360	0.85	357	0.85	
Leu	157	0.37	154	0.37	
lle	929	1.44	930	1.44	trnI-GAU
					trnI-CAU
lle	418	0.65	415	0.64	trn(f)M-CAU
					trnM-CAU
lle	588	0.91	589	0.91	trnM-CAU
Met	571	1	569	1	
Val	479	1.44	481	1.44	trnV-GAC
Val	189	0.57	190	0.57	trnV-UAC
Val	462	1.39	460	1.38	
Val	203	0.61	204	0.61	
Ser	515	1.79	510	1.77	trnS-GGA
Ser	272	0.94	272	0.94	trnS-UGA
Ser	328	1.14	327	1.14	
Ser	156	0.54	160	0.56	
Pro	376	1.45	377	1.46	trnS-GCU
Pro	210	0.81	209	0.81	
Pro	276	1.07	277	1.07	
Pro	172	0.67	168	0.65	trnP-UGG
Thr	519	1.69	518	1.69	
Thr	220	0.72	218	0.71	
Thr	344	1.12	347	1.13	
Thr	147	0.48	146	0.48	trnT-UGU
Ala	588	1.72	591	1.73	
Ala	227	0.66	226	0.66	
Ala	369	1.08	369	1.08	trnG-UCC
Ala	183	0.54	179	0.52	trnA-UGC
Tyr	695	1.61	693	1.61	
Tyr	169	0.39	168	0.39	
Stop	42	1.58	43	1.61	trnY-GUA

Stop	22	0.83	22	0.83	
His	419	1.54	419	1.53	
His	125	0.46	127	0.47	
Gln	643	1.53	640	1.53	
Gln	195	0.47	197	0.47	trnH-GUG
Asn	776	1.54	778	1.54	trnQ-UUG
Asn	235	0.46	235	0.46	
Lys	925	1.42	923	1.41	
Lys	380	0.58	388	0.59	trnN-GUU
Asp	759	1.57	760	1.57	trnK-UUU
Asp	210	0.43	211	0.43	
Glu	927	1.39	931	1.39	
Glu	411	0.61	405	0.61	trnD-GUC
Cys	188	1.43	188	1.44	trnE-UUC
Cys	75	0.57	74	0.56	
Stop	16	0.6	15	0.56	
Trp	414	1	414	1	trnC-GCA
Arg	310	1.27	311	1.27	trnW-CCA
Arg	104	0.42	101	0.41	trnR-ACG
Arg	338	1.38	337	1.38	
Arg	104	0.42	104	0.43	
Ser	342	1.19	343	1.19	
Ser	115	0.4	116	0.4	trnR-UCU
Arg	447	1.82	447	1.83	
Arg	167	0.68	167	0.68	
Gly	525	1.27	526	1.27	trnG-GCC
Gly	192	0.46	192	0.46	trnG-UCC
Gly	605	1.46	605	1.46	
Gly	333	0.8	331	0.8	

[1] RSCU, Relative synonymous codon usage.

Table S7: Details of polymorphic simple sequence repeats (SSRs) in the chloroplast genomes of *D. asper* and *D. japonicus*.

No.	Location[1]	Region[2]	Motif	Repeat number	
				D. asper	*D. japonicus*
1	IGS (*rps16*, *trnQ-UUG*)	LSC	A	10	9
2	IGS (*rps16*, *trnQ-UUG*)	LSC	T	13	11
3	IGS (*psbK*, *psbI*)	LSC	T	11	10
4	IGS (*trnS-GCU*, *trnG-UCC*)	LSC	A	10	9
5	Intron (*atpA*, *atpF*)	LSC	A	20	16
6	Intron (*atpF*, *atpF*)	LSC	T	10	11
7	IGS (*trnT-UGU*, *trnL-UAA*)	LSC	T	11	10
8	IGS (*trnT-UGU*, *trnL-UAA*)	LSC	A	10	12
9	IGS (*trnL-UAA*, *trnL-UAA*)	LSC	A	9	8
10	IGS (*trnF-GAA*, *ndhJ*)	LSC	A	9	8
11	IGS (*trnF-GAA*, *ndhJ*)	LSC	T	11	10
12	Intron (*trnV-UAC*, *trnV-UAC*)	LSC	T	10	9
13	Intron (*rbcL*, *accD*)	LSC	A	8	9
14	IGS (*rbcL*, *accD*)	LSC	T	8	10
15	IGS (*psbE*, *petL*)	LSC	T	20	14
16	IGS (*psbE*, *petL*)	LSC	T	11	9
17	IGS (*clpP*, *clpP*)	LSC	T	9	12
18	IGS (*clpP*, *clpP*)	LSC	T	12	10
19	IGS (*clpP*, *clpP*)	LSC	A	9	10
20	IGS (*clpP*, *clpP*)	LSC	T	11	10
21	IGS (*clpP*, *psbB*)	LSC	A	9	10
22	IGS (*petD*, *rpoA*)	LSC	T	11	10
23	IGS (*rps8*, *rpl14*)	LSC	T	9	11
24	IGS (*rpl14*, *rpl16*)	LSC	A	14	13
25	IGS (*rpl14*, *rpl16*)	LSC	T	10	9
26	IGS (*ccsA*, *ndhD*)	SSC	T	12	10
27	Intron (*ndhD*, *psaC*)	SSC	A	11	9

[1] IGS, intergenic spacer.

[2] LSC, large single copy region; SSC, small single copy region.

Table S8: Details of palindromic repeats in the chloroplast genomes of *D. asper* and *D. japonicus*.

Species	Position	Loop (bp)	Position	Repeat unit length (bp)	Repeat sequence	Region
D. asper *D. japonicus*	IGS *(trnH-GUG, psbA)*	6	IGS *(trnH-GUG, psbA)*	33	TTAAATAAAAAGGGAGCAATAATCCCCCTCTTG	LSC
	IGS *(psbM, trnD-GUC)*	64	IGS *(trnE-UUC, trnT-GGU)*	25	TATTTGGGTTTGGGGTTCGTTCTTT	LSC
	Intron *(petD, petD)*	2	Intron *(trnT-GGU, psbD)*	25	TAAGTGAACTAGATAACCCAGAATC	LSC

Table S9: List of primers used for the amplification of SCAR markers.

Primer name	Primer sequence (5′→3′)	Product size	Position
DAJ-AC_F	GCTAAGTTCTATTGAGGAGGCTCC	185 bp in *D. asper* and 329 bp in *D. japonicus*	*accD*
DAJ-AC_R	CGACATCCTCATCCTCCTCATCGA		
PU-M_F	GGATTTGGGGGCAAACCTGC	256 bp in *P. umbrosa*	*matK*
PU-M_R	GCGTACTACAGAAGGGTTCCTT		

Table S10: Details of germplasm used in this study.

No.	Species	Information collected	Voucher number	DNA barcode analysis	SCAR marker test	Completed chloroplast genome
1	*D. asper*	Goejeong-ri, Cheongju-si, Chungcheongbuk-do, Korea	KIOM201701018837	o	o	o
2		Agricultural Seedling Station, Aewol-eup, Jeju-si, Jeju-do, Korea	KIOM201201004803	o	o	
3		Agricultural Seedling Station, Aewol-eup, Jeju-si, Jeju-do, Korea	KIOM201201004804	o	o	
4		Songming, Yunnan, China	KIOM201501011655	o	o	
5		Hezhang, Bijie, Guiyang, Guizhou, China	KIOM201101004457	o	o	
6	*D. japonicus*	Samsoo-dong, Taebaek-si, Gangwon-do, Korea	KIOM-2016-277	o	o	o
7		Sabuk-ri, Sabuk-eup, Jeongseon-gun, Gangwon-do, Korea	KIOM-2016-269	o	o	
8		Yeongchun-myeon, Danyang-gun, Chungcheongbuk-do, Korea	KIOM201701018775	o	o	
9	*P. umbrosa*	Jeopsan Mt., Geoun-ri, Yeongwol-eup, Yeongwol-gun, Gangwon-do, Korea	KIOM201501014658	o	o	
10		Wangdusan Mt., Chunyang-myeon, Bonghwa-gun, Gyeongsangbuk-do, Korea	KIOM201401010793	o	o	
11		Seolcheon-myeon, Muju-gun, Jeollabuk-do, Korea	KIOM201501012395	o	o	
12		Seorak-myeon, Gapyeong-gun, Gyeonggi-do, Korea	KIOM201301005989	o	o	

Table S11: List of chloroplast genomes downloaded from NCBI for phylogenetic analysis.

No.	Taxon	Order	Family	GenBank accession number
1	*Kolkwitzia amabilis*	Dipsacales	Caprifoliaceae	KT966716.1
2	*Lonicera japonica*			KJ170923.1
3	*Patrinia saniculifolia*			MG517444.1
4	*Tetradoxa omeiensis*		Adoxaceae	KX258653.1
5	*Viburnum utile*			KX792264.1
6	*Adoxa moschatellina*			KX258652.1
7	*Sambucus williamsii*			KX510276.1
8	*Sinadoxa corydalifolia*			KX258651.1
9	*Eleutherococcus senticosus*	Apiales	Araliaceae	JN637765.1
10	*Fatsia japonica*			KR021045.1
11	*Kalopanax septemlobus*			KC456167.1
12	*Metapanax delavayi*			KC456165.1
13	*Panax ginseng*			AY582139.1
14	*Panax notoginseng*			KJ566590.1
15	*Schefflera delavayi*			KC456166.1
16	*Aralia undulata*			KC456163.1
